# Mutations Q93H and E97K in *TPM2* Disrupt Ca-Dependent Regulation of Actin Filaments

**DOI:** 10.3390/ijms22084036

**Published:** 2021-04-14

**Authors:** Małgorzata Śliwinska, Katarzyna Robaszkiewicz, Piotr Wasąg, Joanna Moraczewska

**Affiliations:** Department of Biochemistry and Cell Biology, Faculty of Biological Sciences, Kazimierz Wielki University in Bydgoszcz, Ks. J. Poniatowskiego 12 Str., 85-671 Bydgoszcz, Poland; gosia.sl@ukw.edu.pl (M.Ś.); robkat@ukw.edu.pl (K.R.); piwas@ukw.edu.pl (P.W.)

**Keywords:** thin filament, congenital myopathy, distal arthrogryposis, tropomyosin, homodimers, heterodimers, point mutations, contraction regulation

## Abstract

Tropomyosin is a two-chain coiled coil protein, which together with the troponin complex controls interactions of actin with myosin in a Ca^2+^-dependent manner. In fast skeletal muscle, the contractile actin filaments are regulated by tropomyosin isoforms Tpm1.1 and Tpm2.2, which form homo- and heterodimers. Mutations in the *TPM2* gene encoding isoform Tpm2.2 are linked to distal arthrogryposis and congenital myopathy—skeletal muscle diseases characterized by hyper- and hypocontractile phenotypes, respectively. In this work, in vitro functional assays were used to elucidate the molecular mechanisms of mutations Q93H and E97K in *TPM2*. Both mutations tended to decrease actin affinity of homo-and heterodimers in the absence and presence of troponin and Ca^2+^, although the effect of Q93H was stronger. Changes in susceptibility of tropomyosin to trypsin digestion suggested that the mutations diversified dynamics of tropomyosin homo- and heterodimers on the filament. The presence of Q93H in homo- and heterodimers strongly decreased activation of the actomyosin ATPase and reduced sensitivity of the thin filament to [Ca^2+^]. In contrast, the presence of E97K caused hyperactivation of the ATPase and increased sensitivity to [Ca^2+^]. In conclusion, the hypo- and hypercontractile phenotypes associated with mutations Q93H and E97K in Tpm2.2 are caused by defects in Ca^2+^-dependent regulation of actin–myosin interactions.

## 1. Introduction

**Figure 1:**
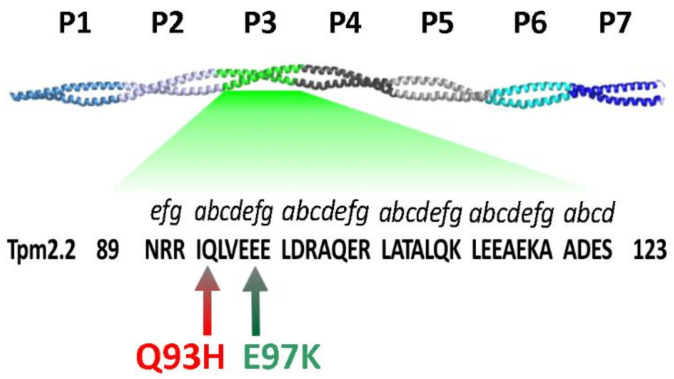
Localization of substitutions Q93H and E97E in the sequence of actin-binding period 3 of Tpm2.2. The coiled coil structure of tropomyosin is illustrated by the 7 Å crystal structure of Tpm1.1 (PDB: 1C1G). Actin-binding periods P1–P7 are marked with different shades of blue and grey, except for P3, which is marked with green. The arrows point to the positions of the substitutions in the sequence (amino acids 89–123) of human Tpm2.2 (NCBI: NP_003280.2). Positions of the amino acids in the heptapeptide repeat (*a–g*) are shown above the amino acid sequence.

Tropomyosin isoforms are proteins associated with most actin filaments present in different compartments of eukaryotic cells. They stabilize the filaments while at the same time acting as gatekeepers that control the access of numerous actin-binding proteins to the filament [1,2]. Tropomyosin is an elongated two-chain coiled coil stabilized by hydrophobic interactions between residues located at the interface between two parallel α-helices. In the coiled coil, strong interchain contacts are possible due to a pattern of seven residues (marked as *abcdefg*) that repeat along the sequence. Because the *a* and *d* residues are mostly hydrophobic, the helical twist allows the hydrophobic residues to lock the chains in a stable dimer. The tropomyosin sequence contains six to seven pseudorepeats between 39 and 41 amino acids long (Figure 1), in which the N-terminal half of the repeat harbors amino acid residues that directly interact with actin. Due to the head-to-tail interactions between adjacent molecules, tropomyosin binds along the actin helix to form uninterrupted cable-like polymers on both sides of the filament [3,4,5,6,7].

Tropomyosin forms either homodimers containing one type of isoform or heterodimers composed of two different polypeptide chains [8]. In skeletal muscle, the contractile thin filaments are regulated by three tropomyosin isoforms. The fast muscle fibers express Tpm1.1 and Tpm2.2, which are products of two different genes, namely *TPM1* and *TPM2*. Tpm1.1 forms both homodimers and heterodimers with Tpm2.2. The latter isoform is also produced in slow muscle fibers where it forms heterodimers with Tpm3.12, the slow fiber-specific isoform encoded by *TPM3* [9,10,11]. Heterodimers differ from homodimers in their thermal stability, actin affinity and regulation of actin–myosin interactions [12,13,14,15], thus formation of heterodimers increases functional diversity among tropomyosin isoforms.

In striated muscle, tropomyosin binds the troponin complex (Tn) and forms the Ca^2+^-dependent regulatory machinery of the thin filament, which controls the ATP-driven cycle of actomyosin cross-bridges. The regulatory mechanism of actin–myosin interactions is best described by a three-state model of thin filament activation, in which the blocked state represents the inactive state, the open state is fully active, and the closed state is intermediate [16]. Structurally, the activation states are represented by different positions of tropomyosin chains on the filament, referred to as B-, C-, and M-state [17]. At rest, when [Ca^2+^] in the sarcoplasm is low, the inhibitory subunit of troponin (TnI) binds to actin and constrains tropomyosin in a blocking position (B-state). In response to muscle fiber activation, sarcoplasmic [Ca^2+^] rises and binds to troponin C (TnC), starting conformational rearrangements in the thin filament which eventually lead to a shift in the position of tropomyosin from B- to C-state. Transition to the third state requires binding of myosin heads to actin and tropomyosin, resulting in a further shift of tropomyosin and full activation of actomyosin interactions (M-state) [18,19].

Precise regulation of the interactions of myosin heads with actin is essential for maintaining the contraction characteristics of different types of muscle. Because tropomyosin is central to the regulatory process, structural changes in tropomyosin isoforms can result in pathological states. In humans, mutations in the tropomyosin gene *TPM1* are associated with hypertrophic and dilated cardiomyopathy—severe heart dysfunctions caused by impaired Ca^2+^-dependent regulation of actomyosin interactions [20,21]. Mutations in *TPM2* and *TPM3* are linked to congenital myopathies (CM) and distal arthrogryposis (DA), clinically and histologically variable disorders of skeletal muscle [21,22,23,24,25]. The mutations are mostly isoform-specific and are randomly distributed along the chains of tropomyosin isoforms, and therefore affect interactions of tropomyosin with different binding partners. Interestingly, mutations which result in opposite phenotypes may be located in close proximity, meaning that functional changes cannot be readily predicted from the position of a mutation. A striking example are substitutions found in a short segment comprising residues 90–97 in all three striated muscle isoforms of tropomyosin, which are linked either to hypo- or hypercontractile phenotypes [20,22,23,24]. This mutational ‘hotspot’ is located within actin-binding period 3, a region which is particularly important for enhancing the actin–myosin interactions [26,27,28].

To better understand the involvement of the N-terminal half of actin-binding period 3 in the regulation of actin–myosin interactions, we compared the effects of two missense mutations in *TPM2* (Figure 1), which cause opposite phenotypes in affected patients. The first, Q93H, was found in CM patients with neonatal hypotonia and respiratory difficulty [22]. This mutation substitutes Gln for His in position *b* of the heptapeptide repeat which, according to the F-actin-tropomyosin atomic model, does not directly interact with actin, but is located between actin-binding residues [29,30,31]. The second mutation, E97K, is a cause of distal arthrogryposis type 1 (DA1), a congenital disorder that involves limb contractures [23]. This mutation reverses the charge of Glu97 located in the *f* position of the heptapeptide repeat (Figure 1) and interacts electrostatically with actin [29,30,31]. We used in vitro assays, which were performed on actin filaments reconstructed with wild type and mutant proteins. Due to the preference of Tpm2.2 to form heterodimers with Tpm1.1 [11,32] and the heterozygosity of both mutations [22,23], the effects of these mutations on the functions of Tpm2.2 homodimer and heterodimers of Tpm2.2 with Tpm1.1 were compared.

We demonstrated for the first time that the mutation Q93H, linked to CM, severely decreased the ability of tropomyosin-troponin to activate interactions between actin and myosin in the presence of Ca^2+^ and rendered the thin filament less sensitive to activating [Ca^2+^]. For substitution E97K, associated with DA, an opposite effect was observed—hyperactivation of the actomyosin interactions and increased sensitivity of the regulatory system to Ca^2+^. Both mutations were dominant, as the presence of mutation in only one chain did not diminish their effects. The mutations tended to decrease the affinity of tropomyosin homo- and heterodimers for actin, but the effects were not severe. Changes in actin affinity did not correlate with Ca^2+^-dependent activation of actomyosin interactions. These observations led to the conclusion that actin-binding period 3 of Tpm2.2 is not critical for tropomyosin interactions with actin, but is strongly involved in Ca^2+^-dependent regulation of actomyosin interactions. 

## 2. Results

### 2.1. Formation of Heterodimers by Wild Type and Mutant Tpm2.2

**Figure 2:**
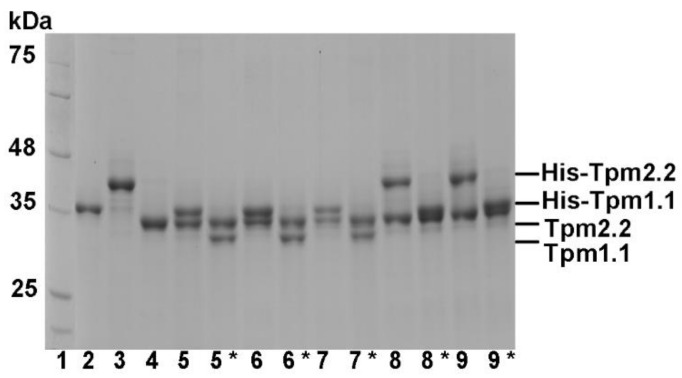
SDS-PAGE of heterodimers after separation on an affinity chromatography column and cleavage with thrombin. Molecular weight standards (lane 1), His-Tpm1.1 (lane 2), His-Tpm2.2 (lane 3), Tpm2.2 (lane 4), His-Tpm1.1/Tpm2.2 (lane 5), Tpm1.1/Tpm2.2 (lane 5 *), His-Tpm1.1/Tpm2.2-Q93H (lane 6), Tpm1.1/Tpm2.2-Q93H (lane 6 *), His-Tpm1.1/Tpm2.2-E97K (lane 7), Tpm1.1/Tpm2.2-E97K (lane 7 *), His-Tpm2.2/Tpm2.2-Q93H (lane 8), Tpm2.2/Tpm2.2-Q93H (lane 8 *), His-Tpm2.2/Tpm2.2-E97K (lane 9), Tpm2.2/Tpm2.2-E97K (lane 9 *). Asterisks mark the untagged heterodimers that were used for the biochemical assays. Samples were separated on a reducing 10% SDS-PAGE gel.

To examine the functional effects of the mutations in tropomyosin variants found in muscle cells, we tested three forms of tropomyosin dimers: (1) Tpm2.2 homodimer with substitutions in both chains; (2) Tpm2.2 heterodimer of wild type and mutant Tpm2.2; and (3) Tpm1.1/Tpm2.2 heterodimer formed by folding wild type or mutant Tpm2.2 with wild type Tpm1.1. The method of heterodimer production is based on His-tag affinity chromatography [13]. Wild type His-tagged Tpm1.1 or Tpm2.2 were mixed with mutant Tpm2.2, heated up to separate the chains, and, after cooling, crosslinked by oxidation of Cys residues and then separated on an affinity column (see Materials and Methods). SDS-PAGE gels of the preparations obtained at subsequent stages of the homo- and heterodimer production are shown in Figure 2. The ratio of Tpm1.1 and Tpm2.2 was estimated by densitometry of the bands after cleavage of the His-tag with thrombin. Because in Tpm2.2 heterodimers with substitution in one chain the bands migrated very close to each other, the ratio was calculated from the band density before removal of the His-tag. Densitometry of the preparations showed that the ratio of tropomyosin chains varied between 0.89 to 1.14 ± 0.04 (upper to lower band ratio), which was close to 1:1. This confirmed that the preparations of heterodimers were homogenous and contained very little, if any, homodimer contamination. Because oxidation of the crosslinked Cys residues requires incubation with DTT at high temperature [15], thermal unfolding-refolding of the tropomyosin coiled coil may lead to an exchange of chains resulting in a mixture of homo- and heterodimers. Therefore, the heterodimers used in the assays were maintained in a crosslinked state. 

### 2.2. Effects of the Mutations Q93H and E97K on Interactions of Tpm2.2 Homo- and Heterodimers with Actin Filament

**Figure 3:**
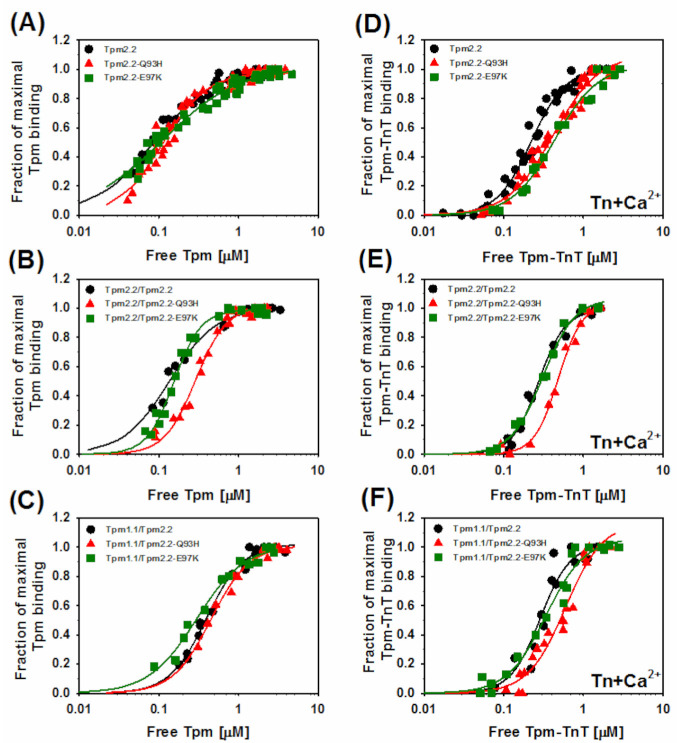
Effects of the substitutions Q93H and E97K in Tpm2.2 on tropomyosin binding to actin alone (**A**–**C**) and in the presence of troponin (Tn) (**D**–**F**). The symbols are: black circles, Tpm2.2; red triangles, Tpm2.2-Q93H; green squares, Tpm2.2-E97K. Conditions: 5 mM imidazole, pH 7.0, 2 mM MgCl_2_, 150 mM NaCl (−Tn) or 300 mM NaCl (+Tn + Ca^2+^). Protein concentrations and normalization method are described in Materials and Methods.

Given the localization of the substitutions Q93H and E97K—either in close vicinity or within the actin-binding site [29,30,31]—changes in the affinity of mutant Tpm2.2 were expected to be one of the most prominent effects of the mutations. For this reason, affinities of wild type and mutant Tpm2.2 in the three dimer forms were examined (Figure 3).

Substitutions Q93H and E97K in homodimers decreased the apparent affinity constant (K_app_) of Tpm2.2 binding to actin alone, but this was not statistically significant. When the substitutions were present in only one Tpm2.2 chain, both mutations decreased affinity, with Q93H having much stronger effect. The presence of Tpm1.1 in one chain lowered the K_app_ value of wild type and mutant tropomyosins. In this form the mutations appeared to have opposite effects—while Q93H tended to decrease, E97K increased the affinity, but once again, the differences were not significant (Table 1). The Hill coefficient (α^H^), which indicates binding cooperativity, was not strongly affected either by mutations or by the variants of heterodimers.

To see how strongly the mutations affected actin affinity to the crosslinked Tpm2.2 homodimer, wild type and mutant homodimers were subjected to chemical oxidation of Cys residues. Crosslinking decreased the actin affinity of all types of tropomyosin, and the mutants had more profound effects than the noncrosslinked variants did. A comparison of the actin binding curves of noncrosslinked and crosslinked homodimers is illustrated in Figure S1. The values of K_app_ obtained for crosslinked wild type Tpm2.2, Tpm2.2-Q93H, and Tpm2.2-E97K were: 7.4 ± 0.5 μM^−1^, 2.9 ± 0.3 μM^−1^, and 5.8 ± 0.6 μM^−1^, respectively. One can therefore conclude that despite being affected by crosslinking, tropomyosin binding to actin was sensitive to the type of tropomyosin chain and specific amino acids located near actin-binding sites.

In order to create conditions suitable for the analysis of Tpm2.2 binding to actin in the presence of troponin and Ca^2+^, salt concentration in the assay buffer was increased. Therefore, the values of K_app_ obtained in the absence and presence of troponin cannot be directly compared. In the presence of troponin, Q93H decreased actin affinity of both types of heterodimers, but E97K had no significant effect (Table 1). Furthermore, in the presence of Tn and Ca^2+^, all variants of tropomyosin bound cooperatively with slight fluctuations in α^H^ but demonstrated no specific trends. 

### 2.3. Effects of Mutations Q93H and E97K on Dynamics of Tropomyosin Interactions with F-actin

Free tropomyosin is easily digested by trypsin, but this process is partially inhibited by F-actin [33]. Because the time course of digestion depends on the tropomyosin sequence [34], the rate of trypsin digestion may be indicative of changes to tropomyosin dynamics due to heterodimer formation or amino acid substitutions. 

**Figure 4:**
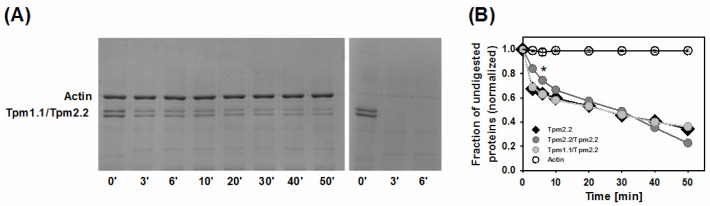
Trypsin digestion of wild type Tpm2.2 homo- and heterodimers. (**A**) Representative polyacrylamide gels showing the digestion time course of heterodimer Tpm1.1/Tpm2.2 in the presence (left panel) and absence (right panel) of F-actin. (**B**) Averaged fraction of undigested F-actin (open circles) in the presence of tropomyosin variants. Fraction of undigested wild type tropomyosin variants bound to F-actin: Tpm2.2 homodimer (black diamonds), crosslinked Tpm2.2/Tpm2.2 (dark grey circles), crosslinked Tpm1.1/Tpm2.2 heterodimer (light grey circles). The points represent the mean ± SE (*n* = 3–5). Statistical analysis was carried out by one-way ANOVA for 6 min of digestion (* *p* ≤ 0.05). Conditions as described in Materials and Methods.

First, we checked if the digestion rate of wild type homodimer Tpm2.2 differed from the digestion rate of the crosslinked wild type homo- and heterodimers. As observed previously [34], free tropomyosin was completely digested within 3 min, but dimers were partially protected by F-actin (Figure 4A). Crosslinking the chains of Tpm2.2 slowed down the initial rate of proteolysis, but crosslinked Tpm1.1/Tpm2.2 heterodimer was digested at a rate indistinguishable from the noncrosslinked homodimer (Figure 4B).

**Figure 5:**
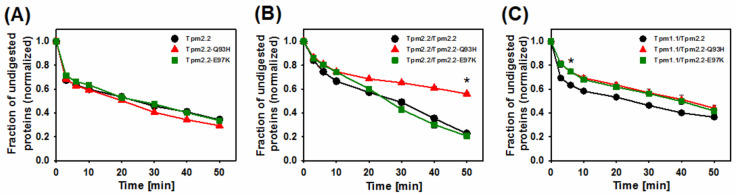
Trypsin digestion rate of tropomyosin homodimers and heterodimers bound to F-actin. Fraction of undigested Tpm2.2 homodimers (**A**), crosslinked Tpm2.2/Tpm2.2 heterodimers (**B**), and crosslinked Tpm1.1/2.2 heterodimers (**C**) in complex with F-actin. Experimental time-courses represent the wild type isoform of each tropomyosin variant (black circles), Q93H mutation (red triangles), and E97K mutation (green squares). Data represent the mean ± SE (*n* = 3–5). Note that the error bars are smaller than the symbols. Statistical analysis was carried out by one-way ANOVA for 6 or 50 min of digestion (* *p* ≤ 0.05). Conditions as described in Materials and Methods.

Further analyses demonstrated that the mutations had no effects on the digestion rate of homodimers with mutation in both chains (Figure 5A). When the mutations were present in only one chain of Tpm2.2, digestion was strongly decreased by Q93H, but not by E97K (Figure 5B). However, in the Tpm1.1/Tpm2.2 heterodimer, both mutations significantly (*p* ≤ 0.05) decreased tropomyosin susceptibility to proteolysis with trypsin (Figure 5C).

### 2.4. Ca^2+^-Dependent Regulation of Actin–Myosin Interactions by Tpm2.2 Mutants

In general, mutations in the tropomyosin genes which result in hypocontractile phenotypes lead to decreased Ca^2+^-dependent activation of actomyosin ATPase and reduced sensitivity to [Ca^2+^]. This contrasts with the hypercontractile phenotypes, which manifest an increased activation of the ATPase and Ca^2+^ sensitivity [21,35]. To test whether this trend is maintained in the case of mutations Q93H and E97K, the regulation of actomyosin activity by tropomyosin-troponin with or without Ca^2+^ was measured. 

**Figure 6:**
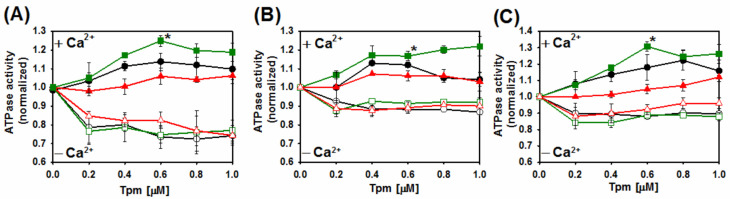
Calcium-dependent regulation of actin-activated myosin S1 ATPase of Tpm2.2 homodimers (**A**), crosslinked Tpm2.2/Tpm2.2 heterodimers (**B**), and crosslinked Tpm1.1/2.2 heterodimers (**C**). The ATPase regulated by Tpm2.2 (black circles), Tpm2.2-Q93H (red triangles), and Tpm2.2-E97K (green squares) in the presence of Tn + Ca^2+^ (closed symbols) and Tn − Ca^2+^ (open symbols) in 30 mM NaCl, 2 mM MgCl_2_, 5 mM imidazole, pH 7.0, 1 mM DTT and either 0.1 mM CaCl_2_ or 0.2 mM EGTA. Protein concentrations: 5 μM F-actin, 1 μM myosin S1, Tn at 1.2 molar excess over Tpm. Data were analyzed using a one-way ANOVA at 0.6 μM tropomyosin (* *p* ≤ 0.05).

First, changes to ATPase activity as a function of tropomyosin-troponin concentration were analyzed in the presence and absence of Ca^2+^ (Figure 6). The results demonstrate that in the presence of Ca^2+^ the substitution Q93H did not activate the interactions between actin and myosin heads. The effect of the mutation was dominant, because the lack of activation was observed in the presence of homodimers and both types of heterodimers. The E97K substitution had an opposite effect, causing hyperactivation of the actomyosin ATPase. The effect of E97K on the regulatory properties of Tpm2.2 was also dominant, again being observed for Tpm2.2 homodimers and both types of heterodimers. Neither mutation significantly disturbed the inhibition process. Interestingly, crosslinked heterodimers had a lower potential to inhibit the ATPase than the noncrosslinked homodimers did. 

**Figure 7:**
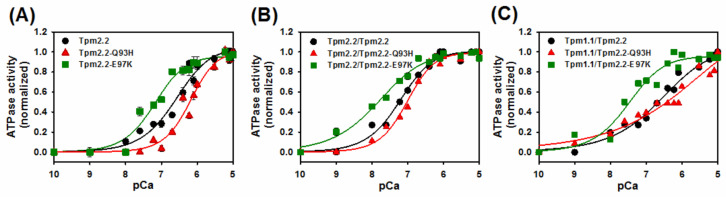
Sensitivity of the actin filaments to increasing [Ca^2+^]. Actomyosin ATPase activity in the presence of Tpm2.2 homodimers (**A**), crosslinked Tpm2.2/Tpm2.2 heterodimers (**B**), and crosslinked Tpm1.1/2.2 heterodimers (**C**). The symbols are: actomyosin S1 ATPase regulation by Tpm2.2 (black circles), Tpm2.2-Q93H (red triangles), and Tpm2.2-E97K (green squares) in complex with troponin. The lines were generated by fitting the experimental points to the Hill equation (Equation (3)). Protein concentrations: 5 μM F-actin, 1 μM myosin S1, 0.6 μM tropomyosin, and 1.0 μM Tn. Other conditions as in Figure 6 legend.

To estimate any effects of mutations on the sensitivity of the tropomyosin-troponin regulatory complex to activating Ca^2+^, ATPase was measured as a function of [Ca^2+^] (Figure 7). In all variants of tropomyosin, the mutation Q93H decreased the sensitivity of the regulatory proteins to [Ca^2+^]. This contrasts with E97K, which increased sensitivity. The parameters of Ca^2+^-sensitivity (Table 2) are expressed as pCa_50_, which is the Ca^2+^ concentration required for a half-maximal switch from full inhibition to maximal activation. A large, one order of magnitude difference was observed between homodimers carrying either Q93H or E97K mutations. Crosslinking the wild type Tpm2.2 chains caused an almost fourfold increase in Ca^2+^-sensitivity, but formation of the crosslinked wild type heterodimer decreased the sensitivity back to the level observed for noncrosslinked homodimer. The presence of Tpm1.1 decreased the sensitivity of both Tpm2.2 mutants, to varying degrees. 

To ensure that, under conditions of the ATPase assay illustrated in Figure 7, tropomyosin-troponin complex bound to actin filaments, we used cosedimentation (not illustrated), which showed that all tropomyosin variants and troponin were bound to F-actin. This confirmed that changes in the Ca^2+^-sensitivity are due to mutation-dependent distortions of the regulatory function of Tpm2.2.

## 3. Discussion

Regulation of skeletal muscle contraction is a sophisticated process which requires cooperation between proteins of the thin filament and myosin heads. Since tropomyosin interacts with actin, troponin, and myosin [29,36,37,38], it is central to the mechanism of contractile regulation. Although a lot of data has accumulated over the years through intense biochemical and structural studies, the regulatory mechanism is not fully understood. These analyses have focused on disease-linked mutations, and have both revealed the molecular basis of muscle disease development and identified tropomyosin regions that are crucial for Ca^2+^-dependent regulation of muscle contraction. The results presented in this work are part of a project which aims to understand the role of actin-binding period 3, a conserved region of tropomyosin that is identical in three isoforms of skeletal muscle tropomyosin. We examined two mutations in *TPM2*, namely Q93H and E97K, which, in spite of being only four residues apart, cause opposite phenotypes. We found that in Tpm2.2 the actin-binding period 3 is genuinely involved in Ca^2+^-dependent regulation of actomyosin interactions, which underlies defects linked to disease-causing mutations. As no studies were previously done to explain the phenotypes associated with these mutations, this study adds information on the molecular bases of hypo- and hypercontractile phenotypes observed in patients carrying mutations in tropomyosin genes. 

Affinity for actin of the Tpm2.2 homo- and heterodimers was analyzed in the presence and absence of troponin—conditions which represent the closed state to the thin filament [29]. The effects of the mutations on the affinity were rather mild, although Q93H was more deleterious than E97K. This agrees with earlier observations that myopathy-related substitution L99M in actin-binding period 3 of Tpm1.1 had no effect on binding to actin in the closed state [39] and that deletion of the whole actin-binding period 3 from Tpm1.1 did not prevent tropomyosin from binding to the filament [40]. A recent high-resolution model of the cardiac thin filament obtained by cryo-electron microscopy [36], together with refinement of tropomyosin contacts with actin by molecular docking [29], offer a platform for structural considerations of the effects the mutations exert on tropomyosin–actin interactions. In the closed state, residue E97—along with R91, E96, and D100 within actin-binding period 3—appear to form an interface that interacts with K326 and K328 on actin. Thus, charge reversal by the substitution E97K should weaken the interactions, which is indeed the case. Although not directly involved in binding to actin, Q93 is located between actin-binding residues R91, E96, and E97. The substitution may therefore destabilize the interface and lead to decreased affinity. 

How susceptible tropomyosin is to trypsinolysis provides insight into any structural heterogeneity of homo- and heterodimers of wild type and mutant Tpm2.2. Free tropomyosin is cleaved very quickly, but is partially protected by F-actin [33,34]. The differences in the proteolysis rate between tropomyosin variants can be explained by their specific dynamics or orientation, which may either expose or hide the peptide bonds recognized by trypsin. One can expect that proteolysis reduces tropomyosin binding, which leads to dissociation of the cut molecule from the filament. Our results showed that crosslinking of Tpm2.2 homodimers decreased the initial digestion rate, but the presence of two different tropomyosin isoforms in heterodimers made the molecule more susceptible to proteolysis, which suggests that the orientation of tropomyosin was changed. In noncrosslinked Tpm2.2 homodimers, neither the presence of Q93H nor E97K changed the dynamics of tropomyosin on actin, but the mutations had different effects on dynamics of heterodimers. This agrees with the results of previous studies, which demonstrated that disease-linked substitutions in both Tpm1.1 and Tpm2.2 differentially affected properties of homo- and heterodimers [15,41]. Since no high-resolution structures of actin filaments with tropomyosin heterodimers are available, further studies are needed to show the exact nature of the structural changes caused by the presence of two different chains in tropomyosin coiled coil. 

We found that effects of mutations on the affinity of Tpm2.2 for actin was dependent on whether that mutation was present in the homo- or heterodimer. This leads to the conclusion that the orientation of actin period 3 must be different in the homo- and heterodimers. This is an interesting observation, because the sequence of actin-binding period 3 is identical in Tpm1.1 and Tpm2.2. Amino acid differences scattered throughout the sequences of Tpm1.1 and Tpm2.2 cause conformational changes, which may be transmitted to actin-binding period 3. In the neighboring actin-binding period 4, isoforms Tpm1.1 and Tpm2.2 harbor five amino acid differences. Because period 4 twists when bound to actin [42], orientation of this segment and the adjacent period 3 may be isoform-dependent. Another possibility is that sequence differences within the C-termini of the isoforms determine structural variations in homo- and heterodimers. Recent structural data obtained by docking tropomyosin segments to F-actin suggest that the structure of the head-to-tail overlap is important for positioning of tropomyosin on the filament [29]. 

The effects of the mutations on the Ca^2+^-dependent regulation of actomyosin ATPase activity provide an excellent explanation of the hypo- and hypercontractile phenotypes observed in patients affected with these mutations. The variants of tropomyosin carrying the substitution Q93H either in one or two chains did not activate the ATPase, and hence were found to be hypocontractile. Conversely, the presence of the substitution E97K resulted in hyperactivation of the ATPase, consistent with the hypercontraction observed in arthrogryposis. In addition, both mutations shifted the Ca^2+^-sensitivity curves in opposite directions; the presence of E97K facilitated the half-maximal activation of the ATPase at a much lower concentration than wild type tropomyosin. As in the presence of Q93H, the ATPase was not activated and increasing Ca^2+^ concentrations merely released the inhibition. Still, much higher concentrations of Ca^2+^ were required for half-release. These results and those of other studies on disease-causing mutations in the three tropomyosin genes give quite a clear picture of the molecular basis of the phenotypes: while increased activation of the actomyosin ATPase and Ca^2+^-sensitivity underlie hypercontractile phenotypes, hypocontractions are caused by reduced activation of the ATPase activation and Ca^2+^-sensitivity [20,21,24,25,39,43]. 

The structural mechanisms of pathological consequences of the substitutions are less clear. The affinity of tropomyosin for actin may contribute to the activation of the thin filament by limiting or facilitating transitions between different activation states. By changing the binding energy, the disease-causing mutations may distort the regulation of actin–myosin interactions [44]. Computational studies on mutation-dependent changes in the energy of actin–tropomyosin interactions suggested that destabilization of tropomyosin–actin contacts in the blocked state shifts the equilibrium between the activation states towards the open state. This facilitates higher activation at lower [Ca^2+^], characteristic of the hypercontractile phenotype [44]. However attractive this model is, our experimental results do not support it; hypercontractile mutations in actin-binding period 3 studied herein had very small effect on the affinity of tropomyosin in the closed state, while a decrease was characteristic for the hypocontractile mutations in all three tropomyosin isoforms [15,39,45]. The recent atomic model of the cardiac thin filament demonstrated that only one tropomyosin chain of tropomyosin coiled coil forms electrostatic contacts with charged residues exposed on the surface of actin [29]. Binding of Ca^2+^ to TnC causes the second chain to swing on the outside of the hinge formed by the tropomyosin–actin interface [29,36]. Thus, the affinity of tropomyosin to actin may not be critical for the equilibrium between the B- and C-state. 

The near atomic structure of the thin filament locates troponin core domain (comprising TnC bound to TnT and TnI) on tropomyosin period 4. The N-terminal domain of TnT extends from the core domain towards the head-to-tail overlap of the opposite tropomyosin chain, therefore one Tn complex embraces two actin chains. The C-terminal domain of TnI extends from the core domain and, at low Ca^2+^ levels, binds to actin and tropomyosin segment comprising actin-binding periods 4 and 3 [36]. By modeling the thin filament in B-state, Lehman’s group identified several electrostatic interactions between the C-terminal domain of TnI and tropomyosin, including E97 and R91 in actin-binding period 3 [46]. Biochemical examination of the effects of mutations within regions of protein interactions can validate this model. In this and the previous work [15,45], we analyzed functional effects of mutations in three tropomyosin isoforms expressed in striated muscle: Tpm1.1, Tpm2.2, and Tpm3.12. Assuming that the electrostatic tropomyosin–TnI interactions stabilize the off state, substitutions of the charged residues should release inhibition. However, our experimental data do not agree with this assumption as neither the E97K charge reversing substitution (in this work) nor the neutralizing substitution R91C [45] impaired inhibition of the actomyosin ATPase activity. One has to note though, that the near-atomic structure [36] and the computational model [29] of the thin filament were obtained for the cardiac tropomyosin isoform Tpm1.1. Since our assays showed that mutations in Tpm2.2 and Tpm3.12 did not interfere with inhibition of the actomyosin ATPase, it is possible that these isoforms interact with TnI differently than Tpm1.1. Such a possibility is supported by the observation that in Tpm1.1 the substitution I92T—located near the tropomyosin–TnI interaction site—enhanced inhibition of the ATPase in the absence of Ca^2+^ [15]. On the other hand, substitutions within actin-binding period 3 may interfere with the activation process. Binding of Ca^2+^ to the N-lobe of TnC opens a hydrophobic patch that attracts the switch peptide of TnI and pulls the C-terminal domain of TnI from actin and tropomyosin [36,47]. Repulsion of the C-terminal domain of TnI by positively charged Lys in mutation E97K might facilitate this process and make the thin filament more sensitive to [Ca^2+^]. In contrast to E97K, the mutations I92T (Tpm1.1), R91C (Tpm3.12), and Q93H (Tpm2.2) shift the filament into a less active state by reducing the thin filament sensitivity to Ca^2+^. Because the three substitutions are located in the tropomyosin segment, which directly contacts TnI, they must interfere with tropomyosin–TnI interactions and affect the on-off switch mechanism. However, at the moment it would be too speculative to propose details of the structural defects which impair activation. 

In conclusion, actin-binding period 3—the inner segment of tropomyosin that is conserved among muscle isoforms of tropomyosin—is crucial for the mechanism of regulation of actomyosin interaction in response to changes in [Ca^2+^]. The phenotypes associated with disease-related mutations are a direct consequence of distortions in this regulatory mechanism. 

## 4. Materials and Methods

### 4.1. Preparation of Muscle Proteins

Fast skeletal actin, myosin, and troponin were isolated from New Zealand rabbit back and hind leg muscle. Fresh meat was a gift from the Department of Pathobiochemistry and Clinical Chemistry, Collegium Medicum in Bydgoszcz. The animals were sacrificed according to the procedures approved by the Committee for Ethical Experiments on Animals of Collegium Medicum. 

Actin was prepared using a standard method described by Spudich and Watt [48]. The concentration of actin was determined from the absorbance at 290 nm using extinction coefficient 0.63 and MW 42,000 for 0.1% actin. Myosin subfragment S1 was prepared by papain digestion according to Margossian and Lowey [49]. Troponin was isolated with the method described by Potter [50]. The concentrations of proteins were determined from the absorbance at 280 nm using extinction coefficients 0.83 and 0.45 and MW 130,000 and 76,000 for 0.1% myosin S1 and troponin, respectively.

### 4.2. Expression and Purification of Recombinant Wild Type and Mutant Tropomyosin Homo- and Heterodimers

Human cDNA Tpm2.2 (NM_003289.4) was synthesized and optimized for bacteria expression by Shanghai ShineGene Molecular Biotech, Inc. The cDNA was cloned into pET11a vector (Novagen Inc., Madison, WI, USA). Wild type human *Tpm2.2* cDNA construct was further used as a template to prepare mutants Gln93His (Q93H) and Glu97Lys (E97K). The substitutions were introduced using PCR-based oligonucleotide-directed mutagenesis kit (Agilent Technologies, Santa Clara, CA, USA). The following forward oligonucleotides were used:

Q93H: 5′ CTGAACCGTCGTATCCACCTGGTTGAAGAAGAAC 3′
E97K: 5′ GTATCCAGCTGGTTGAAAAAGAACTGGACCGTGC 3′
Codons that were mutated are underlined.

The oligonucleotides were synthesized and HPLC purified by the Laboratory of DNA Sequencing and Oligonucleotide Synthesis, Institute of Biochemistry and Biophysics (Warsaw, Poland). Plasmids were transformed into XL-1 supercompetent cells and after DNA isolation (GeneMATRIX Plasmid Miniprep DNA Purification Kit, Eurx, Gdańsk, Poland) substitutions were verified by DNA sequencing in the laboratory mentioned above. Wild type and mutant Tpm2.2 were expressed in *E. coli* BL21(DE3) cells as described before [51]. The concentration of tropomyosin was determined spectrophotometrically at 280 nm using molar extinction coefficient 17,880. 

To produce His-tagged Tpm1.1 (NM_001018005.2) and Tpm2.2, cDNAs encoding human Tpm1.1 and Tpm2.2 were subcloned into pET15b (Novagen Inc.) with HisTag affinity sequence followed by thrombin recognition sequence at the 5′ end. His-Tpm1.1, His-Tpm2.2, and untagged Tpm2.2 were used to produce crosslinked wild type and mutant Tpm1.1/Tpm2.2 heterodimers and crosslinked Tpm2.2/Tpm2.2 heterodimers with mutation in one chain. Proteins were mixed and crosslinked by Cys oxidation which was catalyzed by incubation with 10 mM K_3_Fe(CN)_6_ and 2 μM CuSO_4_ for 3.5 h at 37 °C and then overnight at 4 °C. Homodimers were separated from heterodimers by affinity chromatography on His Select Nickel Affinity Gel (Sigma Aldrich, Saint Louis, MO, USA). The procedures are described in detail in [15].

### 4.3. Actin-Binding Assay

The affinity of wild type and mutant Tpm2.2 for actin was determined using a cosedimentation assay as described before [15]. Briefly, 5 µM filamentous actin was titrated with increasing tropomyosin concentrations (0–5 µM) at room temperature in 5 mM imidazole, pH 7.0, 150 mM NaCl, and 2 mM MgCl_2_—except in the presence of Tn where binding was analyzed in 300 mM NaCl instead. Tn complex, when used, was added at 1.2 M excess over tropomyosin. After 30 min incubation, actin with bound tropomyosin or tropomyosin-troponin was pelleted by ultracentrifugation. Samples obtained from pellets and supernatants were separated on 1% or 12% (in the presence of Tn complex) SDS-PAGE gels. Quantitative analyses of proteins bands were carried out densitometrically. To normalize the data, the densitometric tropomyosin/actin or (tropomyosin + TnT) per actin ratios were divided by the maximal ratios obtained at saturation. Normalized points were drawn versus unbound tropomyosin (or tropomyosin + TnT) concentration measured in supernatants. The experimental points were fit to the Hill equation to obtain the apparent binding constant K_app_ and the Hill cooperativity coefficient α^H^:

(1) $$v={\left(n\times \left[\mathrm{Tpm}\right]\right)}^{\alpha^{H}}\times K_{\mathrm{app}}{}^{\alpha^{H}}/\left(1+{\left[\mathrm{Tpm}\right]}^{\alpha^{H}}\times K_{\mathrm{app}}\right)$$

where: v = fraction of F-actin saturation with tropomyosin, n = maximal saturation of the actin filament with tropomyosin (or tropomyosin-TnT), [Tpm] = concentration of unbound tropomyosin, K_app_ = apparent association constant, α^H^ = Hill cooperativity coefficient. The K_app_ and α^H^ values and standard errors showing deviation of the fitted curve from experimental points were from the statistical data reported by Sigma Plot 12.5 for each binding curve.

### 4.4. Actomyosin MgATPase Activity

Measurements of the regulation of actomyosin S1 ATPase activity by wild type and mutant Tpm2.2 were conducted in 96-well microplates at 22 °C. The samples were in 30 mM NaCl, 2 mM MgCl_2_, 5 mM imidazole, pH 7.0, 1 mM DTT, and either 0.1 mM CaCl_2_ or 0.2 mM EGTA. The reaction was initiated by 5 mM Mg-ATP and terminated after 3 min by 3.3% SDS and 30 mM EDTA. The amount of liberated inorganic phosphate was determined colorimetrically [52].

Tropomyosin-dependent regulation of the ATPase activity was analyzed at constant 5 µM F-actin and 1 µM myosin S1. Tropomyosin concentration was varied between 0–1 µM, troponin was at 1.2-molar excess over tropomyosin. The experimental data were normalized by dividing the activity at each tropomyosin-troponin concentration by the activity of unregulated acto-S1 ATPase (0 µM tropomyosin-troponin). 

Ca^2+^-sensitivity of the actomyosin ATPase activity was measured at Ca^2+^ concentrations increasing from 1 × 10^−10^ to 1 × 10^−3^ M. The proteins were used at the following concentrations: 5 µM actin, 1 µM myosin S1, 0.6 µM tropomyosin, and 1.0 µM troponin. The experimental points were normalized according to the equation:

(2) $$\left(A-A_{\min}\right)/\left(A_{\max}-A_{\min}\right)$$

where: A = ATPase activity at a given point, A_min_ = ATPase activity at 1 × 10^−10^ M Ca^2+^, A_max_ = ATPase activity at 1 × 10^−3^ M Ca^2+^. The values of pCa_50_ were obtained by fitting the experimental points in SigmaPlot 12.5 to the Hill equation:

(3) $$v={\left(A_{\max}\times {\mathrm{pCa}}_{50}\right)}^{\alpha^{H}}\times {\mathrm{pCa}}_{50}{}^{\alpha^{H}}/\left(1+{\mathrm{pCa}}_{50}{}^{\alpha^{H}}\times {\mathrm{pCa}}_{50}\right)$$

Statistical analysis was carried out in SigmaPlot 12.5. One-way ANOVA was used to identify statistical significance of the differences between wild type and mutant Tpm2.2.

### 4.5. Tropomyosin Digestion with Trypsin 

F-Mg-actin was obtained by incubation of G-actin with 0.2 mM EGTA and 0.5 mM MgCl_2_, followed by polymerization with 100 mM NaCl and 2 mM MgCl_2_. Tropomyosin homodimers and heterodimers (2 µM) and 5 µM F-Mg-actin were incubated for 20 min and then digested at room temperature with trypsin (7 μg/mL) in buffer containing 5 mM Hepes pH 7.6, 100 mM NaCl, 5 mM MgCl_2_, and 0.2 mM ATP. At time intervals small aliquots were withdrawn and the reaction was stopped with 2-molar excess of SBTI over trypsin. Samples were separated on 10% polyacrylamide gels using SDS-PAGE electrophoresis. Densities of actin and tropomyosin bands were quantitated using EasyDens software. Statistical analysis was carried out in SigmaPlot 12.5. One-way ANOVA was used to identify statistical significance of the differences between wild type and mutant Tpm2.2.

## Figures and Tables

**Table 1:** Binding parameters of Tpm2.2 homodimers and heterodimers to F-actin in the presence and absence of Tn (Ca^2+^).

Tropomyosin Variants	−Tn		+Tn (Ca^2+^)	
	K_app_ [µM^−1^]	α^H^	K_app_ [µM^−1^]	α^H^
Tpm2.2	11.6 ± 1.9	1.2 ± 0.3	4.5 ± 0.2	2.0 ± 0.2
Tpm2.2-Q93H	9.1 ± 0.7	1.6 ± 0.3	2.4 ± 0.2 *	1.5 ± 0.1
Tpm2.2-E97K	9.1 ± 0.9	0.9 ± 0.1	2.3 ± 0.2 *	1.6 ± 0.2
Tpm2.2/Tpm2.2	7.4 ± 0.5	1.5 ± 0.2	3.7 ± 0.2	2.6 ± 0.3
Tpm2.2/Tpm2.2-Q93H	3.5 ± 0.1 *	2.2 ± 0.2	2.1 ± 0.1 *	2.9 ± 0.3
Tpm2.2/Tpm2.2-E97K	6.5 ± 0.2 *	2.6 ± 0.2	3.2 ± 0.1	2.2 ± 0.2
Tpm1.1/Tpm2.2	2.5 ± 0.1	1.9 ± 0.2	3.5 ± 0.3	2.3 ± 0.5
Tpm1.1/Tpm2.2-Q93H	2.2 ± 0.4	1.8 ± 0.7	1.7 ± 0.2 *	1.8 ± 0.3
Tpm1.1/Tpm2.2-E97K	3.4 ± 0.3	1.4 ± 0.2	2.9 ± 0.2	1.8 ± 0.2

The parameters were obtained by fitting the experimental binding curves shown in Figure 3 to Equation (1). All parameters state the mean ± standard error (SE). Protein binding conditions are described in Materials and Methods. * Statistically significant differences between wild type and mutant tropomyosin (*p* ≤ 0.05). K_app_, apparent affinity constant; α^H^, Hill coefficient.

**Table 2:** Sensitivity of tropomyosin-troponin-regulated myosin S1 ATPase to Ca^2+^ concentrations.

Tropomyosin Variants	pCa_50_ [M]
Tpm2.2	6.6 ± 0.01
Tpm2.2-Q93H	6.2 ± 0.1 *
Tpm2.2-E97K	7.2 ± 0.1 *
Tpm2.2/Tpm2.2	7.2 ± 0.1
Tpm2.2/Tpm2.2-Q93H	7.0 ± 0.1
Tpm2.2/Tpm2.2-E97K	7.8 ± 0.1 *
Tpm1.1/Tpm2.2	6.6 ± 0.1
Tpm1.1/Tpm2.2-Q93H	6.0 ± 0.1 *
Tpm1.1/Tpm2.2-E97K	7.5 ± 0.1 *

The data represent the mean ± SE (*n* = 3–5). * Statistically significant values between wild type and mutant tropomyosin (*p* ≤ 0.05).

## Data Availability

The data presented in this study are available from the authors.

## Supplementary Materials

The following are available online at https://www.mdpi.com/article/10.3390/ijms22084036/s1, Figure S1: Effects of crosslinking on binding of Tpm2.2 to F-actin.

## Abbreviations

CM, congenital myopathy; DA, distal arthrogryposis; Tpm2.2, homodimer of Tpm2.2 isoform; Tpm2.2/Tpm2.2, crosslinked homodimer; Tpm1.1/Tpm2.2, crosslinked heterodimer of two tropomyosin isoforms; DTT, dithiothreitol; SBTI, soy bean trypsin inhibitor; EGTA, Ethylene glycol-bis(2-aminoethylether)-N,N,N0,N0-tetraacetic acid.
