# Supplementary figures and images for: Mutations Q93H and E97K in TPM2 Disrupt Ca-Dependent Regulation of Actin Filaments
Source: Int J Mol Sci. 2021 Apr 14;22(8):4036. doi: 10.3390/ijms22084036 (PMC8070786; doi:10.3390/ijms22084036)

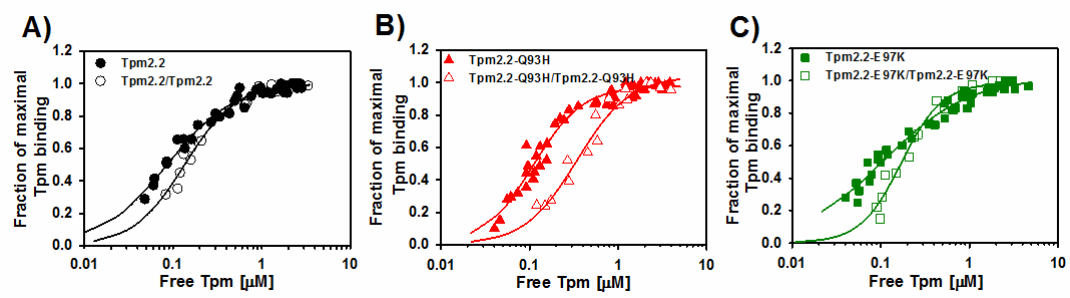

Figure S1. Effects of crosslinking on binding of Tpm2.2 to F-actin.

Supplement: Supplementary file 1 [file ijms-22-04036-s001.pdf]
